# A validation study of data in the National Tonsil Surgery Register in Sweden: high agreement with medical records ensures that data can be used to monitor clinical practices and outcomes

**DOI:** 10.1186/s12874-021-01467-8

**Published:** 2022-01-07

**Authors:** Filip Lundström, Erik Odhagen, Fredrik Alm, Claes Hemlin, Pia Nerfeldt, Ola Sunnergren

**Affiliations:** 1 Department of Otorhinolaryngology, Linköping University Hospital, Linköping, Sweden; 2 Department of Biomedical and Clinical Sciences, Linköping University, Linköping, Sweden; 3 Department of Otorhinolaryngology, Södra Älvsborgs Hospital, Borås, Sweden; 4 Department of Research, Education and Innovation, Södra Älvsborgs Hospital, Borås, Sweden; 5 School of Health Sciences, Faculty of Medicine and Health, Örebro University, Örebro, Sweden; 6 Department of Otorhinolaryngology, Aleris Sollentuna, Sollentuna, Sweden; 7 Department of Otorhinolaryngology, Karolinska University Hospital, Stockholm, Sweden; 8 Department of Clinical Science, Intervention and Technology (CLINTEC), Karolinska Institutet, Stockholm, Sweden; 9 Department of Otorhinolaryngology, Region Jönköping County, Jönköping, Sweden

**Keywords:** Tonsil surgery, Tonsillectomy, Tonsillotomy, Medical quality register, Health quality improvement

## Abstract

**Background:**

The ambition of the National Tonsil Surgery Register in Sweden (NTSRS) is to improve otorhinolaryngological care by monitoring trends in the clinical practices, complications, and outcomes of tonsil surgery. The NTSRS collects data from both surgeons and patients and provides the participating clinics with daily updated data on a publicly available website. On the website, national and local results can be compared and monitored. The use of NTSRS data necessitates that the data is valid, but the NTSRS has not yet been validated. With approximately half of the registered patients responding to the postoperative questionnaires, an analysis of responders and non-responders is also necessary. The aim of this study was to assess the criterion validity of NTSRS data. Another aim was to compare the characteristics and rates of complications between postoperative questionnaire responders and non-responders.

**Methods:**

Data in the NTSRS were compared with data in electronic medical records. The 200 most recent surgeries, up to 31 Dec 2019, in each of 11 surgical units were included. Criterion validity was analysed in terms of observed agreement, Cohens kappa, Gwet’s AC_1_, and positive and negative agreement. The sign test was used to analyse systematic differences between the NTSRS and the medical records. Comparisons of rates between groups were made with Fisher’s exact test, the chi-square test, and Fisher’s non-parametric permutation test.

**Results:**

A total of 1991 registrations were included in the study. All variables showed very high observed agreement ranging from 0.91 to 1.00, and all variables had AC_1_ values corresponding to almost perfect agreement. The analysis of questionnaire responders and non-responders showed no statistically significant differences regarding age, indication, or type of surgery. The proportion of women was higher in the responder group. The rate of reoperation due to bleeding was higher in the responder group, but there were no differences regarding other complications.

**Conclusions:**

The results of this study show that data in the NTSRS have criterion validity. The NTSRS is thus well suited for monitoring the clinical practices and outcomes of tonsil surgery. The quality of the data also implies that the registry can be used in both clinical improvement projects and research.

## Background

The National Tonsil Surgery Register in Sweden (NTSRS) was established in 1997 by the Swedish Association for Otorhinolaryngology Head and Neck Surgery. The aim was to improve Ear-, nose- and throat (ENT) care by monitoring trends in the clinical practices, complications, and outcomes of tonsil surgery. The NTSRS aims to include all tonsil surgeries performed for benign indications. The NTSRS was revised to the current version in 2009. Participation in the NTSRS is voluntary, with a majority (82% in 2019) of Swedish surgical centres reporting to the register. A comparison with the Swedish National Patient Register (NPR) showed that in the last decade, approximately 80% of all tonsil surgeries in Sweden have been included [1]. All public and private healthcare providers in Sweden are obliged by law to report the number of performed tonsil surgeries to the NPR. By the end of 2019, > 100,000 surgeries had been registered since the revision in 2009. In the NTSRS, the surgeon registers a perioperative form with information on age, sex, indication, surgical method, surgical technique for dissection and haemostasis, and postoperative bleeding occurring during the hospital stay. At 30 days and at 6 months after surgery, the patient is requested to fill in questionnaires with patient-reported outcome measures (PROMs). The 30-day questionnaire focuses on postoperative complications and recovery, and the 6-month questionnaire focuses on the success of the surgery (symptom relief). The mean response rate from 2009 to 2018 was 55% for the 30-day questionnaire and 46% for the 6-month questionnaire. A detailed description of the variables and the data collection procedure is available in English at the NTSRS website [1].

The NTSRS can be used as a benchmark to identify differences in clinical outcomes between surgical units and evaluate the effect of quality improvement projects. The registry provides the participating clinics with daily updated data, both raw and processed, via a publicly available website [2]. The results can thus be monitored longitudinally and compared with those of other surgical units. The register should function as a stimulator of competition to achieve best practice [3]. A health care register, such as the NTSRS, does also provide data to measure value in health care [4] by monitoring the most important outcomes of tonsil surgery. Data from the NTSRS have been used in several scientific publications to describe clinical practices, compare surgical techniques, and evaluate postoperative complications [5–10]. NTSRS data have also been used in clinical quality improvement projects [11]. Such use of health care register data necessitates that the data be reliable and valid [12, 13], but the NTSRS has not yet been validated. Thus, the NTSRS needs to be validated in a large-scale, multicentre, systematically conducted study.

A method widely used in quality register validation is to compare register data with expert reviewed data from medical records [14, 15]. This is also the recommended method for validation according to the Swedish Association of Local Authorities and Regions, funders of the Swedish quality registers [16]. The results of such an audit of a health register should both be published to substantiate register quality, and used to improve data quality in the register [17]. There are only two national tonsil surgery registers in the world, the Swedish and the Norwegian. The Norwegian register was launched in 2017 and structured as a copy of the NTSRS. In 2019, The Norwegian Tonsil Surgery Register published the results of an agreement analysis (including analysis of observed agreement, Cohen’s kappa and Gwet’s AC1 coefficients) of the register database and medical records [15]. This study showed almost perfect agreement but the study was a single-centre study, with a limited number of patients, and only variables related to the surgery were analysed. It would strengthen both tonsil surgery registers if the results could be reproduced in a multi-centre study, with a substantially larger study population also including outcome PROM data. With approximately half of the registered patients responding to the PROM questionnaires, an analysis of responders and non-responders regarding both general characteristics and outcomes is also necessary.

The aim of this study was to evaluate the criterion validity of data in the NTSRS by comparison with data in medical records. Other aims were to gain a better understanding of potential weaknesses regarding the validity of NTSRS variables, and to compare the characteristics and rates of complications in patients who did and did not respond to the 30-day postoperative PROM questionnaire.

## Methods

### Study design

A retrospective comparison of data in the NTSRS and data in electronic medical records (EMRs) was conducted for the 200 most recently performed surgeries, up to 31 Dec 2019, in each of 11 surgical units. The set goal of 2200 patients at 11 surgical centres was decided with three intentions. The first intention was to include a composition of surgical centres representative of the NTSRS, the second intention was to include enough patients at each centre to ensure generalizability, and the third intention was to capture rare events. As clinical practice constantly evolves, we decided to include the 200 most recent surgeries at each unit rather than a random sample to assure that the data analysed in this study reflected today clinical practice and data quality. The choice to include the most recent surgeries rather than a random sample made it impossible, beforehand, to ascertain that the case mix of the studied cohort would be perfectly matched with the total NTSRS cohort. This was a calculated risk, but based on our professional knowledge of Swedish tonsil surgery practice and our choice to include a wide variety of surgical units we assumed that the case mix of the study cohort would be satisfactory comparable with the NTSRS cohort. In 2019, seven university clinics, 20 county hospitals, 10 rural hospitals and 15 private units participated in the NTSRS. With three university clinics, four county hospitals, two rural hospitals and two small private units included in this study, the sample was considered a good representation of Swedish tonsil surgical units.

It was acknowledged in the planning phase that some of the data in the NTSRS might be difficult to validate against medical records as many NTSRS variables are not registered in Swedish EMRs. Therefore some of the NTSRS variables had to be left out of the study. Two examples were “For how many days after surgery did you take painkillers?” and “How many days after surgery did you start eating regular food?”. The steering committee of the NTSRS chose the variables of the study based on professional knowledge of what type of information could be expected to be found in Swedish medical records. The selected variables with definitions are presented in Table 1.

**Table 1:** Variables with comments and definitions

Perioperative data	Definitions and comments
Indication	Snoring/upper airway obstruction/tonsil hypertrophy, recurrent tonsillitis, chronic tonsillitis, peritonsillitis, systemic complications to tonsillitis, other^a^
Type of surgery	Tonsillectomy, tonsillectomy with adenoidectomy, tonsillotomy, tonsillotomy with adenoidectomy
Surgical technique, cold surgery	Cold dissection with cold haemostasis (i.e., no electrosurgical instruments used)
Level of care	Outpatient or inpatient care
Tonsillectomy á chaud	Immediate surgery due to acute infection
Postoperative bleeding	Bleeding that required an intervention: return to theatre, administration of anti-haemorrhagic drugs, or a blood transfusion before discharge after index surgery
30-day PROM questionnaire	Definitions and comments
Contact due to bleeding	Medical records: Any type of registered contact (telephone, outpatient visit, readmission) due to bleeding occurring after discharge from index surgery
	NTSRS: A yes to the question: “Have you contacted medical care due to bleeding from the throat?”
Admission due to bleeding	Medical records: Readmission due to bleeding occurring after discharge from index surgery
	NTSRS: A yes to the question: “Have you been admitted to hospital due to bleeding from the throat?”
Reoperation due to bleeding	Medical records: Reoperation under full anaesthesia due to bleeding occurring after discharge from index surgery
	NTSRS: A yes to the question: “Was another surgery performed due to bleeding?”
Contact due to pain	Medical records: Any type of registered contact (telephone, outpatient visit, readmission) due to pain occurring after discharge after index surgery
	NTSRS: A yes to the question: “Have you contacted medical care because of pain after the surgery?”

*Abbreviations*: *NTSRS* National Tonsil Surgery Register in Sweden

^a^Other = all other indications than the before mentioned

There was only one exclusion criterion for the study: patients were excluded if they had a permanent residence outside the catchment area (i.e. health care region) of the surgical unit. The reason was that information on postoperative complications would be impossible to find in the medical records used by the operating clinic, as there is no national medical record system in Sweden. In Sweden, health care is provided by 21 health care regions. The EMRs used for data retrieval in this study were common for the respective health care region. This means that the EMRs covered provided care in quite large geographical areas, including all hospitals and all ENT departments belonging to the health care region in question. Primary care EMRs were not used for data retrieval in this study. For PROM data analysis, only patients who responded to the 30-day questionnaire were included.

Previous studies based on the NTSRS have shown that hot (electrosurgical) surgical techniques, used either for dissection or for haemostasis, carry a higher risk for postoperative bleeding after tonsillectomy [7, 8]. Cold (cold steel instruments) techniques have the lowest risk for bleeding, and there is no difference in risk between different electrosurgical techniques [7, 8]. Therefore, the NTSRS decided to present composite data with all techniques reclassified as cold or hot on the NTSRS website. Therefore, surgical techniques were, in this study, dichotomized as either cold (cold dissection with cold haemostasis) or not cold.

### Data collection

The source data in this study was the medical records (which in all cases were electronic medical records) and the NTSRS database. A duplicate of the NTSRS database (the validation database - VDB) was constructed to record the data from the medical records. Seven members (“monitors”) of the NTSRS steering committee participated in data retrieval from the medical records. Behind a secure web login, the monitors entered data directly into the VDB via predefined categories in dropdown lists or checkboxes. The EMRs were accessed at each surgical unit through a computer with access to the data server of the health care region. With the aim of achieving a fair and common assessment of the information in the medical records and analogous registering of data in the VDB, a monitor’s manual was constructed. The medical records were only accessed after obtaining written consent and a signed agreement with the head of the department at each surgical unit. The VDB also allowed additional information to be registered, with the aim of better understanding possible inconsistencies between the NTSRS and the medical records. One example when additional information was valuable was if a patient reported an “admission due to bleeding” to the NTSRS without being admitted according to the medical records. In such cases, the additional information might explain why the patient had misinterpreted the question. Such information could be an admission due to pain or that the patient stayed for overnight observation the first night after surgery due to unexpected peroperative bleeding. Another example was a patient that reported a yes to the NTSRS question “Was another surgery performed due to bleeding” without any corresponding information being found in the EMRs. If such a patient had information in the EMR of a surgical procedure performed in local anaesthesia this information would have been recorded in the VDB. This additional information will be presented in the results section, as appropriate.

Mandatory variables (= no missing data) in the NTSRS are date of surgery, age, gender, indication and type of surgery. Data for all these variables must be entered into the data base to create a valid entry. In the same way age, gender, dates and codes for type of surgery and indication (for all visits and hospitalizations) are mandatory in the EMRs. For the EMRs this means that the only variable where missing data is possible is surgical technique. Naturally, the absence of a registered complication in the EMR is regarded as a non-event and not as missing data.

The registration and collection of data from the medical records were performed in a two-step process. When all registrations were completed, a first mismatch analysis was performed. All variables in the study were nominal scale and thus could only match or not match. All monitors were notified (by e-mail) of the mismatching data at the unit that they had monitored, and each monitor received a data file from the VDB with the mismatching data points. The monitor then double-checked the medical records to ensure that the registration in the VDB was correct. The monitors were at all times blinded to the actual data in the NTSRS. The VDB was stored at the data server of Centre of Registers Västra Götaland and accessible to the monitors via a personal and secure web log- in. The NTSRS data base was stored in the same data server as the VDB but NTSRS data was only accessible to the statisticians. All medical records were electronic, stored at the data server of the respective health care region, and at all surgical units accessible to the monitors via a personal and secure at site log-in.

### Statistical analysis

The distributions of variables are given as numbers and percentages for categorical variables and as the mean and standard deviation (SD) and median (min-max) for continuous variables.

Criterion validity refers to the analysis of agreement (as described below) between two independent data sources, with the medical records considered as non-reference standard. Criterion validity was further explored by the careful descriptive assessment of mis-matching data-points in source data (including the full medical notes). The results of this meticulous evaluation of discrepant data were reported in descriptive text to provide a good understanding of potential weaknesses in criterion validity of the NTSRS variables.

The criterion validity, reflected by the agreement between the data in the NTSRS and the medical records was analysed in terms of observed agreement, Cohen’s kappa, Gwet’s AC_1_ [18], and positive and negative agreement [19]. As many of the variables in the NTSRS have a skewed trait distribution, the kappa is at risk of yielding artificially low coefficients. Under such circumstances, the AC_1_ is a better estimate of the agreement than Cohen’s kappa, as AC_1_ is not influenced by unbalanced trait prevalence. The kappa and AC_1_ coefficients were interpreted as follows: ≤0.20, slight agreement; 0.21–0.40, fair agreement; 0.41–0.60, moderate agreement; 0.61–0.80, substantial agreement; and ≥ 0.81, almost perfect agreement. As we couldn’t assume that the medical records, used for comparison with the NTSRS, were always correct we had to consider medical record data as a non-reference standard [19]. Therefore, the estimates were called positive- and negative agreements even though they were calculated with the same formula as sensitivity and specificity [19]. Systematic differences between the NTSRS and the medical records were analysed with the sign test. The sign test is a conditional test that only considers the non-match registrations of the NTSRS and the medical records. No adjustments were made for multiple comparisons.

Rates were compared between groups by Fisher’s exact test for dichotomous variables and the chi-square test for non-ordered categorical variables. Fisher’s non-parametric permutation test was used for continuous variables. All significance tests were two-sided and conducted with a 5% significance level. SAS software version 9.4 (SAS Institute, Cary, NC, USA) was used for all statistical analyses.

## Results

The intention was to include 2200 patients in the project. After the exclusion criterion was applied, 1991 patients remained in the study population. For comparison, 11,226 patients were registered in the NTSRS in 2019, of which 1514 patients were included in the study population. Thus, the study population accounted for 13,5% of the NTSRS 2019 cohort. The composition of the whole study population was largely comparable to that of the 2019 NTSRS cohort (Table 2).

**Table 2:** General characteristics of the study population and a comparison with the NTSRS cohort for 2019

Variable	Study population (*n* = 1991)	NTSRS 2019^a^ (*n* = 9712)
Age, years		
Mean (SD)	11.7 (12.5)	13.6 (12.5)
Median (Min-max)	6 (0.9; 81.1)	7.5 (1; 83)
Sex, n (%)		
Male	1082 (54.3%)	4858 (50.0%)
Female	909 (45.7%)	4854 (50.0%)
Level of care, n (%)		
Outpatient	1507 (78.9%)	7659 (81.4%)
Inpatient	403 (21.1%)	1746 (18.6%)
Main indication, n (%)		
Snoring/upper airway obstruction/tonsil hypertrophy	1437 (72.2%)	6004 (61.8%)
Recurrent tonsillitis	272 (13.7%)	1633 (16.8%)
Peritonsillitis	99 (5.0%)	474 (4.9%)
Chronic tonsillitis	136 (6.8%)	1397 (14.4%)
Systemic complication to tonsillitis	3 (0.2%)	9 (0.1%)
Other indication	44 (2.2%)	195 (2.0%)
Type of surgery, n (%)		
Tonsillectomy	639 (32.1%)	3835 (39.5%)
Tonsillectomy with adenoidectomy	266 (13.4%)	1353 (13.9%)
Tonsillotomy	120 (6.0%)	444 (4.6%)
Tonsillotomy with adenoidectomy	966 (48.5%)	4080 (42.0%)

^a^Patients in the study population who underwent surgery in 2019 (*n* = 1514) were subtracted from the cohort in this table

Due to the non-mandatory nature of some of the variables in the NTSRS, missing data occurred. Another reason for missing data was lack of information in the medical records. The numbers and percentages of missing data points for the non-mandatory variables in the NTSRS and EMRs are presented in Table 3.

**Table 3:** The number of missing data points for the non-mandatory variables in the NTSRS and the EMRs

Variable	Study population (n)	Missing data, total n (%)	Missing data, NTSRS n (%)	Missing data, EMR n (%)
**Perioperative form**				
Level of care (outpatient)	1991	81 (4,1%)	81 (4,1%)	NA
Surgical technique (cold/cold)	1991	147 (7,4%)	90 (4,5%)	57 (2,9%)
Tonsillectomy á chaud	1991	710 (35,7%)	710 (35,7%)	NA
Postoperative bleeding	1991	211 (10,6%)	211 (10,6%)	NA
**30-day PROM questionnaire**				
Contact due to bleeding	1037	6 (0,6%)	6 (0,6%)	NA
Admission due to bleeding	1037	43 (0,4%)	43 (0,4%)	NA
Reoperation due to bleeding	1037	138 (13,3%)	138 (13,3%)	NA
Contact due to pain	1037	19 (1,8%)	19 (1,8%)	NA

*Abbreviations*: *NTSRS* National Tonsil Surgery Register in Sweden, *EMR* Electronic medical record, *NA* not applicable

The date of surgery differed by less than 7 days in 98% of the registrations and by more than 21 days in only 1%. All other variables showed very high observed agreement ranging from 0.91 to 1.00, and all variables had AC_1_ values corresponding to almost perfect agreement (Table 4). The kappa values, as expected due to the skewed trait distributions, showed much greater variability (Table 4). Seven of the variables (where 4 were different indications) had positive agreement < 0.80, while all variables had negative agreement ≥0.92 (Table 4).

**Table 4:** Analyses of agreement between data in the NTSRS and the medical records. No adjustments were made for multiple comparisons

Variable	Totaln	Match n (%)	NTSRS no EMR no n (%)	NTSRS yes EMR yes n (%)	Non- Match n (%)	NTSRS yes EMR no n (%)	NTSRS no EMR yes n (%)	Observed agreement (95% CI)	Positive agreement	Negative agreement	Kappa (95% CI)	AC_1_ (95% CI)	Systematic differences *p*-value^b^
**Perioperative form**													
Indication (all indications)	1991	1814 (91.1%)			177 (8.9%)			0.91 (0.90–0.92)			0.80 (0.78–0.83)	0.90 (0.89–0.92)	
*Snoring*^a^	1991	1906 (95.7%)	514 (25.8%)	1392 (69.9%)	85 (4.3%)	45 (2.3%)	40 (2.0%)	0.96 (0.95–0.97)	0.97	0.92	0.89 (0.87–0.92)	0.93 (0.91–0.94)	0.66
*Recurrent tonsillitis*	1991	1870 (93.9%)	1658 (83.3%)	212 (10.6%)	121 (6.1%)	60 (3.0%)	61 (3.1%)	0.94 (0.93–0.95)	0.78	0.97	0.74 (0.70–0.79)	0.92 (0.91–0.93)	1.00
*Chronic tonsillitis*	1991	1895 (95.2%)	1806 (90.7%)	89 (4.5%)	96 (4.8%)	47 (2.4%)	49 (2.5%)	0.95 (0.94–0.96)	0.64	0.97	0.62 (0.55–0.69)	0.94 (0.93–0.96)	0.92
*Peritonsillitis*	1991	1981 (99.5%)	1888 (94.8%)	93 (4.7%)	10 (0.5%)	6 (0.3%)	4 (0.2%)	0.99 (0.99–1.00)	0.96	1.00	0.95 (0.91–0.98)	0.99 (0.99–1.00)	0.75
*Systemic complication*	1991	1985 (99.7%)	1983 (99.6%)	2 (0.1%)	6 (0.3%)	1 (0.1%)	5 (0.3%)	1.00 (0.99–1.00)	0.29	1.00	0.40 (0.01–0.78)	1.00 (0.99–1.00)	0.22
*Other indication*	1991	1955 (98.2%)	1929 (96.9%)	26 (1.3%)	36 (1.8%)	18 (0.9%)	18 (0.9%)	0.98 (0.98–0.99)	0.59	0.99	0.58 (0.46–0.71)	0.98 (0.97–0.99)	1.00
Level of care (outpatient)	1910	1865 (97.6%)	394 (20.6%)	1471 (77.0%)	45 (2.4%)	36 (1.9%)	9 (0.5%)	0.98 (0.97–0.98)	0.99	0.92	0.93 (0.91–0.95)	0.96 (0.95–0.97)	<.0001
Type of surgery (all types)	1991	1928 (96.8%)			63 (3.2%)			0.97 (0.96–0.98)			0.95 (0.94–0.96)	0.96 (0.95–0.97)	
*TE*	1991	1972 (99.0%)	1344 (67.5%)	628 (31.5%)	19 (1.0%)	11 (0.6%)	8 (0.4%)	0.99 (0.99–0.99)	0.99	0.99	0.98 (0.97–0.99)	0.98 (0.98–0.99)	0.65
*TEA*	1991	1953 (98.1%)	1703 (85.5%)	250 (12.6%)	38 (1.9%)	16 (0.8%)	22 (1.1%)	0.98 (0.97–0.99)	0.92	0.99	0.92 (0.89–0.94)	0.98 (0.97–0.98)	0.42
*TT*	1991	1965 (98.7%)	1868 (93.8%)	97 (4.9%)	26 (1.3%)	23 (1.2%)	3 (0.2%)	0.99 (0.98–0.99)	0.97	0.99	0.87 (0.83–0.92)	0.99 (0.98–0.99)	<.0001
*TTA*	1991	1948 (97.8%)	995 (50.0%)	953 (47.9%)	43 (2.2%)	13 (0.7%)	30 (1.5%)	0.98 (0.97–0.98)	0.97	0.99	0.96 (0.94–0.97)	0.96 (0.94–0.97)	0.014
Surgical technique (cold/cold)	1854	1759 (94.9%)	1484 (80.0%)	275 (14.8%)	95 (5.1%)	73 (3.9%)	22 (1.2%)	0.95 (0.94–0.96)	0.93	0.95	0.82 (0.79–0.86)	0.93 (0.91–0.94)	<.0001
Tonsillectomy á chaud	1281	1278 (99.8%)	1244 (97.1%)	34 (2.7%)	3 (0.2%)	2 (0.2%)	1 (0.1%)	1.00 (0.99–1.00)	0.97	1.00	0.96 (0.91–1.00)	1.00 (0.99–1.00)	1.00
Postoperative bleeding	1780	1761 (98.9%)	1752 (98.4%)	9 (0.5%)	19 (1.1%)	12 (0.7%)	7 (0.4%)	0.99 (0.98–0.99)	0.56	0.99	0.48 (0.28–0.68)	0.99 (0.98–0.99)	0.36
**30-day PROM questionnaire**													
Contact due to bleeding	1031	1008 (97.8%)	960 (93.1%)	48 (4.7%)	23 (2.2%)	16 (1.6%)	7 (0.7%)	0.98 (0.97–0.99)	0.87	0.98	0.79 (0.71–0.88)	0.97 (0.96–0.99)	0.093
Admission due to bleeding	994	984 (99.0%)	947 (95.3%)	37 (3.7%)	10 (1.0%)	9 (0.9%)	1 (0.1%)	0.99 (0.98–1.00)	0.97	0.99	0.88 (0.80–0.95)	0.99 (0.98–1.00)	0.021
Reoperation due to bleeding	899	892 (99.2%)	883 (98.2%)	9 (1.0%)	7 (0.8%)	7 (0.8%)	0 (0.0%)	0.99 (0.98–1.00)	1.00	0.99	0.72 (0.52–0.92)	0.99 (0.99–1.00)	0.016
Contact due to pain	1018	922 (90.6%)	861 (84.6%)	61 (6.0%)	96 (9.4%)	65 (6.4%)	31 (3.0%)	0.91 (0.89–0.92)	0.66	0.93	0.51 (0.42–0.59)	0.88 (0.86–0.91)	0.0007

*Abbreviations*: *NTSRS* National Tonsil Surgery Register in Sweden, *EMR* Electronic medical records, *TE* Tonsillectomy, *TEA* Tonsillectomy with adenoidectomy, *TT* Tonsillotomy, *TTA* Tonsillotomy with adenoidectomy, *PROM *patientreported outcome measure

^a^I.e. snoring/upper airway obstruction/tonsil hypertrophy

^b^Systematic differences, *p*-values, sign test. The sign test uses only information from patients in the non-match group

Regarding the *indication*, 177 (9%) non-matches were observed. Seventy-one of these had multiple indications for surgery according to the medical records, including the indication registered in the NTSRS. Sixty-six patients had a specific non-match between *recurrent* and *chronic tonsillitis*. Thus, 40 out of 1991 (2.0%) of the patients had an unexplainable non-match regarding the indication for surgery.

The most common problem encountered for the variable *level of care* (2.4% mismatch) was that planned outpatient surgery that, according to the medical records, was converted to inpatient surgery was registered as outpatient surgery in the NTSRS.

The observed agreement for surgical technique was 95%, and the kappa and AC_1_ showed almost perfect agreement. Nevertheless, there was a systematic significant difference between the NTSRS and the medical records, with 73 cold surgeries registered in the NTSRS that could not be verified in the medical records.

*Postoperative bleeding* showed high observed agreement, a low kappa but a high AC_1_, and low positive agreement. Only 9 postoperative bleeding events were recorded in both the NTSRS and medical records, while 12 were recorded in the NTSRS only and 7 in the medical records only.

Although the observed agreement *for contact due to bleeding* was high, the kappa showed good agreement, and the AC_1_ showed almost perfect agreement, there were still some important inconsistencies. Seven patients had, according to the medical records, been in contact with the healthcare system without reporting this in the NTSRS questionnaire. On the other hand, 16 patients reported, in the questionnaire, contact that could not be found in the medical records. The medical records revealed that of these 16 patients, two suffered postoperative bleeding that required some type of intervention before discharge from the primary surgery, and nine had documented contact either due to pain or another unspecified reason after discharge from the index surgery.

For *admission due to bleeding*, the observed agreement was 99%, and both the kappa and the AC_1_ showed almost perfect agreement. Only one patient had a registered admission due to bleeding without reporting this in the NTSRS 30-day PROM questionnaire. Nine patients reported admission due to bleeding without corresponding data in the medical records. According to the medical records, one patient had visited the emergency department with a bleeding throat but chose to leave the hospital even though admission was recommended for observation. Another patient had an outpatient visit due to bleeding but was not admitted overnight. One patient experienced postoperative bleeding before discharge after the index surgery, underwent reoperation due to the bleeding and was observed overnight. One patient was observed overnight after a planned outpatient surgery due to anaesthesia-related problems. Two patients were readmitted due to pain after discharge from the primary surgery. Three patients had no information on any contact or complications registered in the medical records. The fact that these 9 patients reported *admission due to bleeding* in the NTSRS without being admitted due to bleeding according to the medical records resulted in a significantly higher rate in the NTSRS than in the medical records (*p* = 0.021).

Regarding *reoperation due to bleeding*, both the observed agreement and the AC_1_ were very high. The kappa was lower but still showed substantial agreement. Nine patients had matching data regarding reoperation, but 7 patients reported reoperation in the NTSRS 30-day questionnaire without supporting information in the medical records. Of these 7 patients who did not have a recorded reoperation, two underwent reoperation under full anaesthesia before they were discharged after the index surgery. Another two underwent intervention with the use of bipolar diathermy under local anaesthesia after discharge from the primary surgery. This variable showed low positive agreement and a statistically significant difference between the NTSRS and the medical records (*p* = 0.016), with a higher rate in the NTSRS.

The variable *contact due to pain* had the lowest observed agreement, kappa and AC_1_ of all the 30-day PROM questionnaire variables. Of the non-matches, 31 patients had a contact in their medical records that was not reported in the NTSRS questionnaire. On the other hand, 65 patients reported a contact that could not be found in the medical records. Of these, 30 patients had some kind of contact recorded in their medical record not interpreted by the monitor as primarily due to pain but instead due to other postoperative problems. The positive agreement was low, and there was a statistically significant difference between the NTSRS and the medical records (*p* = 0.0007), with a higher rate in the NTSRS.

### Comparison of responders and non-responders to the 30-day NTSRS PROM questionnaire

The analysis was based on 1037 responders and 954 non-responders. This means that the response rate in the studied cohort was 52.1%. For comparison the response rate in the total NTSRS cohort for 2019 was 50.9%. No statistically significant difference was found in terms of age, indication, type of surgery or level of care (Table 5). There was a statistically significant difference in the sex distribution (*p* = 0.0022), with a higher proportion of women in the responder group (Table 5).

**Table 5:** Comparison of responders and non-responders to the 30-day NTSRS PROM questionnaire. Data from medical records only. No adjustments were made for multiple comparisons

Variable	Responders (*n* = 1037)	Non-responders (*n* = 954)	*p*-value
Sex, n (%)			
Male	529 (51.0%)	553 (58.0%)	
Female,	508 (49.0%)	401 (42.0%)	0.0022
Age at surgery, years			
Mean (SD)	11.5 (12.7)	10.9 (12.2)	0.27
Median (min-max)	6 (0–79)	6 (0–81)	
Indication, n (%)			0.48
Snoring	730 (70.4%)	702 (73.6%)	
Recurrent tonsillitis	149 (14.4%)	124 (13.0%)	
Chronic tonsillitis	81 (7.8%)	57 (6.0%)	
Peritonsillitis	48 (4.6%)	49 (5.1%)	
Systemic complications to tonsillitis	4 (0.4%)	3 (0.3%	
Other indication	25 (2.4%)	19 (2.0%)	
Level of care, n (%)			0.60
Outpatient surgery	792 (76.4%)	738 (77.4%)	
Inpatient surgery	245 (23.6%)	216 (22.6%)	
Type of surgery, n (%)			0.19
Tonsillectomy	343 (33.1%)	293 (30.7%)	
Tonsillectomy with adenoidectomy	153 (14.8%)	119 (12.5%)	
Tonsillotomy	50 (4.8%)	50 (5.2%)	
Tonsillotomy with adenoidectomy	491 (47.3%)	492 (51.6%)	
Complications, n (%)			
Contact due to bleeding	55 (5.3%)	46 (4.8%)	0.70
Admission due to bleeding	38 (3.7%)	35 (3.7%)	1.00
Reoperation due to bleeding	9 (0.9%)	1 (0.1%)	0.030
Contact due to pain	94 (9.1%)	104 (10.9%)	0.19

Regarding the rates of *contact due to bleeding*, *readmission due to bleeding*, and *contact due to pain,* no differences were found between responders and non-responders to the 30-day PROM questionnaire. There was a significantly higher rate of patients who underwent reoperation due to bleeding in the responder group (Table 5).

## Discussion

With few exceptions, this study shows that the data in the NTSRS have criterion validity. This is a prerequisite if quality register data is to be used to monitor and improve medical care [12, 17]. Based on the AC_1_ values, it can be concluded that the agreement was almost perfect for all variables (range 0.84 to 1.00). The kappa values showed almost perfect or substantial agreement for 16 out of 20 variables. Furthermore, the analysis of responders and non-responders to the 30-day questionnaire showed that women were more prone to respond than men. There were no differences in outcome between patients who did and did not respond with the exception that responders had a significantly higher rate of reoperation due to bleeding. This study establishes that the NTSRS is suited for monitoring clinical practices and outcomes of tonsil surgery. Thus, the registry can be used in both clinical improvement projects and research.

The results in this study are in accordance with those of a similar study from the Norwegian tonsil surgery register [15]. The Norwegian study was a single-centre study conducted on the first 137 consecutive patients ever registered in that registry, while the present study was a much larger (1991 patients) multicentre study. It could be argued that the good results shown in the Norwegian study, at least in part, could be due to dedicated surgeons in a pioneering clinic. It is therefore reassuring that the present study on the NTSRS, operational for almost 25 years, yielded basically the same good results as the Norwegian study.

In both the present study and the Norwegian study, most of the discrepancies regarding the variable *indication for surgery* were found for recurrent tonsillitis and chronic tonsillitis. There are at least two possible explanations. First, there is no code for recurrent tonsillitis in the International Classification of Disorders (ICD) codebook. While the NTSRS allows the surgeon to separate these two indications, the medical records based on the ICD only allow chronic tonsillitis. This may entice the surgeon to register chronic tonsillitis in the medical records even though the patient suffers from recurrent tonsillitis. Second, there is no generally accepted definition for either of these two indications, and there is also a probable overlap between the conditions. It would be appropriate for future versions of the ICD to include a specific code for *recurrent tonsillitis,* as it is a common condition encountered in clinical practice. Studies on the clinical characteristics of these types of infectious tonsillitis are needed to determine the potential differences.

The *surgical technique* showed substantial or almost perfect agreement (kappa, 0.82; AC_1_, 0.93), which indicates that the data regarding surgical techniques in the NTSRS is valid. However, it is important to note that 5.1% of the registrations were non-matches. The most common non-matches (3.9%) were NTSRS cold technique registrations that were contradicted by the medical records. This means that the positive effects of cold techniques found on bleeding complications have not been exaggerated in NTSRS data [7, 8]. As many NTSRS-based clinical improvement projects have been (and hopefully will be) conducted with the aim of increasing the number of surgeries performed using cold techniques, this problem needs to be communicated with surgeons to ensure that registrations in the NTSRS are correct.

The variable *postoperative bleeding*, which is recorded by the surgeon, is intended to capture bleeding that occurs after extubation but before the patient is discharged from the index surgery. In total, 28 such events were recorded in the NTSRS, the medical records or both (Table 4). It is undoubtedly problematic that only 9 were found in both. The reason why 7 events were recorded only in the medical records is probably that the surgeon registers the perioperative data immediately after surgery, while bleeding often occurs somewhat later. Capturing these events in such cases would require the surgeon to return to and change the registration. It is more puzzling why 12 events were captured only in the NTSRS. One explanation could be that surgeons are not aware of the NTSRS definition of postoperative bleeding. According to the NTSRS instructions, only postoperative bleeding that occurs after the patient is extubated should be registered. It is possible that some surgeons register unexpected bleeding occurring before the patient is extubated. Such events would not be identified in the medical records by the monitor and not registered in the VDB. It must be acknowledged that this is an NTSRS variable that in its present form must be interpreted with caution due to suboptimal criterion validity.

Although the statistical analysis showed almost perfect agreement (AC_1_) for all 30-day PROM variables, there were still some notable discrepancies between the NTSRS and the medical records. Unlike the perioperative form where the surgeon can learn about the definitions on the NTSRS webpage, no such information is available to the patients, a circumstance that may increase the risk of misunderstandings and affect criterion validity. The questions in the PROM questionnaire were constructed in 2009 with the intention of collecting data on very specific events. The definitions of these events (as presented in Table 1) have never been publicly shared (e.g., with patients). The variables *admission due to bleeding* and *reoperation due to bleeding* can be taken as examples, as both showed statistically significant differences in rates between the NTSRS and the medical records. Nine patients reported admission without being admitted, and 7 patients reported reoperation without undergoing reoperation. The majority of these patients had a complication but not the complication that the question was intended to capture. The consequence is that the NTSRS is at risk of overestimating the rates of these complications. One explanation for this over-reporting may be that patients have problems understanding the questions or cannot find a question that asks about their specific experience. The results must be taken seriously as they indicate suboptimal criterion validity. By cross-tabulation and statistical analysis of different alternative interpretations of the PROM variables, it was ensured that no alternative interpretation of the questions resulted in better agreement.

One of the 30-day PROM variables stood out with only moderate agreement according to the kappa statistic: c*ontact due to pain*. The major reason for this result is probably a limitation of the study methodology and design. Sweden does not have a national medical record system, and patients may have contacted their general practitioner or the web- and telephone-accessible National Health Care guide (www.1177.se) instead of the operating clinic. Such contact would not be recorded in the medical records that were accessible to the monitors. Differences in the routine to register telephone contact (i.e., some centres may not register such contact in their medical records) at the surgical centres might also be a factor. It is therefore possible that this variable suffers from false low agreement due to missing data. This also means that the difference in rates between the NTSRS and the medical records should be interpreted with caution.

Comparison of the responders and non-responders showed that women were more prone to respond to the questionnaire than men. There was also a significant difference between the responders and non-responders in the variable *reoperation due to bleeding,* with a higher rate of reoperation in the responder group. One possible explanation is that reoperation is so traumatizing that it triggers the patient to respond to the questionnaire. In contrast, no such differences were found in *contact due to bleeding*, *contact due to pain* or *readmission due to bleeding*. The conclusion is that even though the response rate for the 30-day NTSRS questionnaire is slightly above 50%, the rates can be considered representative for the whole population with the exception of *reoperation due to bleeding*.

There are many quality aspects on quality registers [3, 12, 17] and the NTSRS is in a good position to fulfil many of these. The NTSRS have a high coverage and completeness [1], and it is unique as one of only two national tonsil surgery quality registers. It must also be considered consistent as it has been in use for more than a decade without major changes in the most important variables, allowing for longitudinal analysis. The results of this study now adds that the NTSRS data is valid. The NTSRS also fulfils the quality criterion of usefulness; through the register website surgical centres can benchmark best practice and engage in competition to achieve best practice. The outcome measures, both complications and efficacy are relevant both to the patients and the medical care system as they can serve as markers for value in health care [4].

### Strengths and limitations

The multicentre design ensures that the whole spectra of surgical units in Sweden are represented. The large number of included patients indicates that the study sample does not differ in any essential aspect from the NTSRS, and the sample size is well above the number needed for generalizability.

In both the NTSRS and the medical records, data were provided by hundreds of different surgeons (the same clinics and clinicians report both to the NTSRS and the medical records). For the medical records, data were also transferred by the monitors into the VDB*.* These circumstances imply a risk for affecting data quality. Several conditions can on good grounds be assumed to have counteracted this potential weakness. The surgeons that reported to the register had access to the registry guidelines through the NTSRS website, and the carefully constructed monitor manual aimed to ensure that the data collection from the medical records and the registration in the VDB were performed in a similar manner by all the monitors.

The study design has some limitations that probably negatively affected the results. As mentioned before, the monitors only had access to the medical records of the surgical unit and ENT units in the catchment area (i.e. health care region). There are several other health care services (non ENT) available for counselling and guidance, with medical records that were not available to the monitors. Also, patients that had to seek medical care due to a complication while travelling outside their health care region during the postoperative convalescence would also be missed.

Another limitation of this study (and the NTSRS in general) regards timeliness. In quality register methodology timeliness refers to the time lapse from an event to the registration of that event in the data base. The longer the time lapse, the greater the risk that incorrect data is entered. There are at least three problems with timeliness that may negatively affect the data quality in the NTSRS and EMRs and thereby also the results of this study. First, there is no de facto dead-line for the surgeons when it comes to register data in either the EMR or NTSRS. Based on our experience and knowledge however, we assume that most registrations in both the NTSRS and EMRs are made within minutes or hours after the surgery. Second, the NTSRS database does not contain data regarding time from surgery to the registration in the NTSRS meaning that the impact of timeliness cannot be evaluated in this study. Third, the PROM questionnaire analysed in this study is sent out 30 days after surgery, and the patient is expected to give correct answers regarding events that may have occurred several weeks earlier. We acknowledge that NTSRS data collection can be improved regarding timeliness, and the most effective strategy would be to link data directly from medical records to the NTSRS. Another alternative would be to link data from another health register such as the NPR. At present, the NPR cannot be used as it is only updated once a year. Other weaknesses with both EMRs and the NPR are that many of the NTSRS variables (including PROM data) are not registered in either EMRs or the NPR.

## Conclusion

This study establishes that the NTSRS is well suited for monitoring clinical practices and outcomes of tonsil surgery and that the registry can be used in both clinical improvement projects and research. The study also shows that even though approximately half of the patients did not respond to the 30-day PROM questionnaire, the data can be considered representative of the whole population. However, it cannot be ignored that this study shows that there is room for improvement. The careful analysis of each variable indicated that patients sometimes did not seem to understand the questions the way they were intended. This indicates that criterion validity of the NTSRS variables can be improved, for example by providing both surgeons and patients with better information. In the future, the questionnaire could be improved with explanatory text for each question. Additionally, an increased number of questions might be beneficial for capturing negative events not covered by the current questionnaire.

## Data Availability

Data from the NTSRS is regulated by Swedish law. Research using NTSRS data may be carried out following approval by an ethics committee. Please contact the Centre of Registers Västra Götaland in Sweden for more information (registercentrum@vgregion.se).

## Abbreviations

NTSRS: National Tonsil Surgery Registry in Sweden
EMR: Electronic medical records
PROM: Patient-reported outcome measures
VDB: Validation database
TE: Tonsillectomy
TEA: Tonsillectomy with adenoidectomy
TT: Tonsillotomy
TTA: Tonsillotomy with adenoidectomy

## References

[1] The National Tonsil Surgery Register in Sweden, https://ton.registercentrum.se/in-english/the-national-tonsil-surgery-register/p/HJV8b8hV. Accessed 30 Nov 2021.

[2] The National Tonsil Surgery Register in Sweden, https://ton.registercentrum.se/statistik/statistik/p/SkphnLnE. In Swedish. Accessed 30 Nov 2021.

[3] McNeil JJ, Evans SM, Johnson NP, Cameron PA (2010). Clinical-quality registries: their role in quality improvement. Med J Aust.

[4] Porter ME (2010). What is value in health care?. N Engl J Med.

[5] Hessén Söderman AC, Ericsson E, Hemlin C, Hultcrantz E, Månsson I, Roos K, Stalfors J (2011). Reduced risk of primary postoperative hemorrhage after tonsil surgery in Sweden: results from the National Tonsil Surgery Register in Sweden covering more than 10 years and 54,696 operations. Laryngoscope..

[6] Sunnergren O, Hemlin C, Ericsson E, Hessén-Söderman AC, Hultcrantz E, Odhagen E, Stalfors J (2014). Radiofrequency tonsillotomy in Sweden 2009-2012. Eur Arch Otorhinolaryngol.

[7] Söderman AC, Odhagen E, Ericsson E, Hemlin C, Hultcrantz E, Sunnergren O, Stalfors J (2015). Post-tonsillectomy haemorrhage rates are related to technique for dissection and for haemostasis. An analysis of 15734 patients in the National Tonsil Surgery Register in Sweden. Clin Otolaryngol.

[8] Lundström F, Stalfors J, Østvoll E, Sunnergren O (2020). Practice, complications and outcome in Swedish tonsil surgery 2009-2018. An observational longitudinal national cohort study. Acta Otolaryngol.

[9] Alm F, Stalfors J, Nerfeldt P, Ericsson E (2017). Patient reported pain-related outcome measures after tonsil surgery: an analysis of 32,225 children from the National Tonsil Surgery Register in Sweden 2009-2016. Eur Arch Otorhinolaryngol.

[10] Elinder K, Söderman AC, Stalfors J, Knutsson J (2016). Factors influencing morbidity after paediatric tonsillectomy: a study of 18,712 patients in the National Tonsil Surgery Register in Sweden. Eur Arch Otorhinolaryngol.

[11] Odhagen E, Sunnergren O, Söderman AH, Thor J, Stalfors J (2018). Reducing post-tonsillectomy haemorrhage rates through a quality improvement project using a Swedish national quality register: a case study. Eur Arch Otorhinolaryngol.

[12] Solomon DJ, Henry RC, Hogan JG, Van Amburg GH, Taylor J (1991). Evaluation and implementation of public health registries. Public Health Rep.

[13] Sorensen HT, Sabroe S, Olsen J (1996). A framework for evaluation of secondary data sources for epidemiological research. Int J Epidemiol.

[14] Varmdal T, Mathiesen EB, Wilsgaard T, Njølstad I, Nyrnes A, Grimsgaard S, Bønaa KH, Mannsverk J, Løchen ML (2021). Validating acute myocardial infarction diagnoses in National Health Registers for use as endpoint in research: the Tromsø study. Clin Epidemiol.

[15] Wennberg S, Karlsen LA, Stalfors J, Bratt M, Bugten V (2019). Providing quality data in health care - almost perfect inter-rater agreement in the Norwegian tonsil surgery register. BMC Med Res Methodol.

[16] Swedish Association of Local Authorities and Regions, https://skr.se/download/18.71ae53ab179a30d0f2d5ad/1621943497406/Valideringshandboken-nationella-kvalitetsregister.pdf. In Swedish. Accessed 30 Nov 2021.

[17] Govatsmark RE, Sneeggen S, Karlsaune H, Slørdahl SA, Bønaa KH (2016). Interrater reliability of a national acute myocardial infarction register. Clin Epidemiol.

[18] Gwet KL. Computing inter-rater reliability and its variance in the presence of high agreement. Br J Math Stat Psychol. 2008;61:29–48. 10.1348/000711006X126600 18482474

[19] Guidance for industry and FDA staff statistical guidance on reporting results from studies evaluating diagnostic tests. U.S. Department of health and human services, food and drug administration, center for devices and radiological health, diagnostic devices branch, division of biostatistics, office of surveillance and biometrics. Document issued on: March 13, 2007. Available online at: https://www.fda.gov/regulatory-information/search-fda-guidance-documents/statistical-guidance-reporting-results-studies-evaluating-diagnostic-tests-guidance-industry-and-fda.

